# The current landscape of coronavirus-host protein–protein interactions

**DOI:** 10.1186/s12967-020-02480-z

**Published:** 2020-08-18

**Authors:** Laure Perrin-Cocon, Olivier Diaz, Clémence Jacquemin, Valentine Barthel, Eva Ogire, Christophe Ramière, Patrice André, Vincent Lotteau, Pierre-Olivier Vidalain

**Affiliations:** 1 CIRI, Centre International de Recherche en Infectiologie, Univ Lyon, Inserm, U1111, Université Claude Bernard Lyon 1, CNRS, UMR5308, ENS de Lyon, 69007 Lyon, France; 2 UMR Processus Infectieux en Milieu Insulaire Tropical, Université de La Réunion, CNRS, 9192 INSERM U1187, IRD 249, Plateforme de Recherche CYROI, Sainte Clotilde La Réunion, France; 3 Laboratoire de Virologie, Hôpital de la Croix-Rousse, Hospices Civils de Lyon, Lyon, France

**Keywords:** SARS-CoV-2, Coronavirus, Interactome, Virus-host interactions, Protein–protein interactions

## Abstract

In less than 20 years, three deadly coronaviruses, SARS-CoV, MERS-CoV and SARS-CoV-2, have emerged in human population causing hundreds to hundreds of thousands of deaths. Other coronaviruses are causing epizootic representing a significant threat for both domestic and wild animals. Members of this viral family have the longest genome of all RNA viruses, and express up to 29 proteins establishing complex interactions with the host proteome. Deciphering these interactions is essential to identify cellular pathways hijacked by these viruses to replicate and escape innate immunity. Virus-host interactions also provide key information to select targets for antiviral drug development. Here, we have manually curated the literature to assemble a unique dataset of 1311 coronavirus-host protein–protein interactions. Functional enrichment and network-based analyses showed coronavirus connections to RNA processing and translation, DNA damage and pathogen sensing, interferon production, and metabolic pathways. In particular, this global analysis pinpointed overlooked interactions with translation modulators (GIGYF2-EIF4E2), components of the nuclear pore, proteins involved in mitochondria homeostasis (PHB, PHB2, STOML2), and methylation pathways (MAT2A/B). Finally, interactome data provided a rational for the antiviral activity of some drugs inhibiting coronaviruses replication. Altogether, this work describing the current landscape of coronavirus-host interactions provides valuable hints for understanding the pathophysiology of coronavirus infections and developing effective antiviral therapies.

## Background

Like other viruses, coronaviruses are obligate parasites and have evolved a swarm of molecular interactions for hijacking the cellular machinery to replicate. Among those, the subset of physical interactions between viral and cellular proteins—usually referred to as the virus-host interactome—is playing a key role [1]. Mapping these protein–protein interactions (PPIs) has proven extremely useful for an intimate comprehension of viral replication cycles, shedding light on the molecular modules used by viruses to replicate. Virus-host interactomics also helps to understand how viruses are detected by the immune system but also escape immune defense through the evolution of countermeasures. Finally, interactome data can be used to identify and prioritize valuable cellular targets for developing antiviral drugs as previously exemplified [2, 3].

Along with genomic sequences and viral protein structures, interactome data are now considered as basic pieces of information for characterizing a virus at the molecular level. Tremendous efforts have been made to characterize the emerging SARS-CoV-2 (Severe acute respiratory syndrome coronavirus 2), a positive-strand RNA virus of the *Coronaviridae* family, identified as the etiological agent of the ongoing COVID-19 respiratory disease pandemic. In a recent report, viral proteins from the SARS-CoV-2 were individually expressed in human cells and targeted host proteins were identified by affinity purification and mass spectrometry [3]. This first virus-host interactome of the SARS-CoV-2 provided essential information on the pathways targeted by this emerging pathogen, and allowed the authors to propose a list of antiviral drug candidates to be tested. Although this represents an important step in our understanding of this virus, it is known that a single interactomic study cannot offer a comprehensive picture of a virus-host interactome [4]. Indeed, despite the use of top-notch technologies by skilled operators, each dataset contains a substantial level of unidentified interactions and artifacts that is inherent to the technology and bias the results. For instance, protein complex analysis by mass spectrometry does not distinguish direct and indirect virus-host interactions and is usually well-complemented by other technological approaches for detecting binary PPIs such as yeast two-hybrid or protein complementation assays. For these different reasons, mapping the interactome of SARS-CoV-2 can be considered as a work in progress.

As a contribution to this effort, we used an orthogonal approach by looking at virus-host interactions already reported for other coronaviruses. This compendium of data gathered from literature was used to identify both overlapping and complementary interactions to build the framework of a generic coronavirus-host interactome. Although each coronavirus is expected to have evolved specific interactions accounting for host range specificity and pathogenesis, a majority of coronavirus-host PPIs are most likely shared across multiple species considering the high level of conservation of the coronavirus replication machinery [5]. Gathering interactomic data from several related viruses is an efficient way to fill in the blanks from literature and identify cellular pathways and complexes that are common coronaviruses targets. Although a proof of concept of this approach was recently established [5], we were able to retrieve 10 times more interactions from literature to assemble an unmatched collection of coronavirus-host interactions. In addition, we identified PPIs that could explain the antiviral activity of approved drugs previously characterized as coronaviruses inhibitors, thus strengthening their interest against SARS-CoV-2.

## Methods

### Virus-host interaction data collection

PubMed database was interrogated to collect virus-host interaction data for the following coronaviruses: HCoV-NL63, HCoV-229E, HCoV-HKU1, HCoV-OC43, MERS-CoV, SARS-CoV, SARS-CoV-2, TGEV, PRCV, PEDV, MHV, IBV, and PDCoV. The following query sentence was used: “virus name”[Title/Abstract] AND (bind*[Title/Abstract] OR interact*[Title/Abstract]). After careful analysis of the retrieved abstracts, 112 publications explicitly reporting physical interactions between viral and host proteins were selected. These publications were analyzed by at least two curators to determine, according to the EMBL-EBI ontology nomenclature for molecular interactions, which methods were used to characterize the reported interactions. Collected information were gathered in a single data file (Additional file 1: Table S1). All human interactors were identified by their UniProt gene name.

### KEGG pathway enrichment analysis

The list of host proteins interacting with coronavirus proteins was submitted by their UniProt identifier to the Functional Annotation Tool of the online knowledge base DAVID Bioinformatics Resources 6.8, NIAID/NIH [6]. Statistical enrichments in KEGG pathway annotations [7] were calculated by the Functional Annotation Tool, using *Homo sapiens* as background, with EASE score threshold (Fisher Exact Statistics, referring to one-tail Fisher Exact Probability Value used for gene-enrichment analysis) set to 0.01 and a count threshold of 5 genes/pathway for short lists of proteins (below 500) or 15 for longer lists. The protein list was considered to be significantly associated (enriched) with a pathway when Benjamini–Hochberg adjusted p-value was below 0.05.

### Interactions with metabolic pathways

The complete list of human genes associated to “Metabolic pathways” in KEGG was retrieved through DAVID Bioinformatics Resources 6.8, NIAID/NIH (https://david.ncifcrf.gov/kegg.jsp?path=hsa01100$Metabolic%20pathways&termId=550028675&source=kegg). This list was compared to the list of host proteins interacting with coronaviruses to identify overlaps. In total, 62 host genes were present in the two lists and were manually clustered according to the specific pathways they belong to using KEGG hierarchical annotation.

### Metascape analysis

Metascape is a web tool designed to integrate multi-platform OMICs data [8], and was used to interrogate the human interactome with the list of host proteins interacting with coronaviruses. Analysis parameters set by default on Metascape website were applied (“Express analysis” settings). PPIs from the human interactome were retrieved from three databases: BioGrid, InWeb_IM and Omni-Path (Min Network Size = 3, Max Network Size = 500). Densely connected regions were extracted by Metascape using the MCODE algorithm. Finally, GO Enrichment analysis (“GO Biological Process”; integrated to Metascape) was applied to each MCODE component independently, and the best-scoring term by p-value has been retained as the functional description of the corresponding component.

### Host proteins interacting with antiviral drugs

To identify host proteins interacting with drugs showing some antiviral activity against coronaviruses, we used the Drug Repurposing Hub database [9]. The database was downloaded (v03/24/2020), and filtered for drugs reported to inhibit coronaviruses in four large-scale screenings [10–13]. Host proteins interacting with these drugs were compared to the list of host proteins interacting with coronaviruses to identify overlaps.

## Results and discussion

### Gathering coronavirus-host interactions from literature

Several members of the *Coronaviridae* family are pathogenic in human and animals, and represent a threat for public health and livestock. To date, seven coronaviruses have been reported to infect human (Human coronaviruses; HCoVs). HCoV-NL63 and HCoV-229E (from the α genus) and HCoV-HKU1 and HCoV-OC43 (from the β genus) are responsible for common cold. In addition, three β coronaviruses, MERS-CoV (Middle East respiratory syndrome-related coronavirus), SARS-CoV and SARS-CoV-2, are associated to life-threatening respiratory diseases in human. Important animal coronaviruses include but are not limited to TGEV (Transmissible Gastroenteritis Virus, α genus), PRCV (Porcine Respiratory Coronavirus, α genus), PEDV (Porcine Epidemic Diarrhea Virus, α genus), MHV (Murine Hepatitis Virus, β genus), IBV (Infectious Bronchitis Virus, γ genus), and PDCoV (Porcine Deltacoronavirus, δ genus). The genome of coronaviruses is a positive-strand RNA with two-third at the 5′ end occupied by the overlapping open reading frames ORF1a and b that encode non-structural proteins (nsPs). The genes encoding structural proteins and a variable number of accessory factors are nested at the 3′ end of the genome. Viral genomes are directly translated by host cell’s ribosomes into a large polyprotein encoded by the ORF1a/b gene. This polyprotein is cleaved into 16 non-structural proteins (nsP1-16), except for IBV and PDCoV where nsP1 is missing. Most of these nsPs assemble into a complex that replicates viral genome and synthesizes subgenomic mRNA from the other genes to express structural proteins S, E, M and N and the additional accessory factors.

**Fig. 1:**
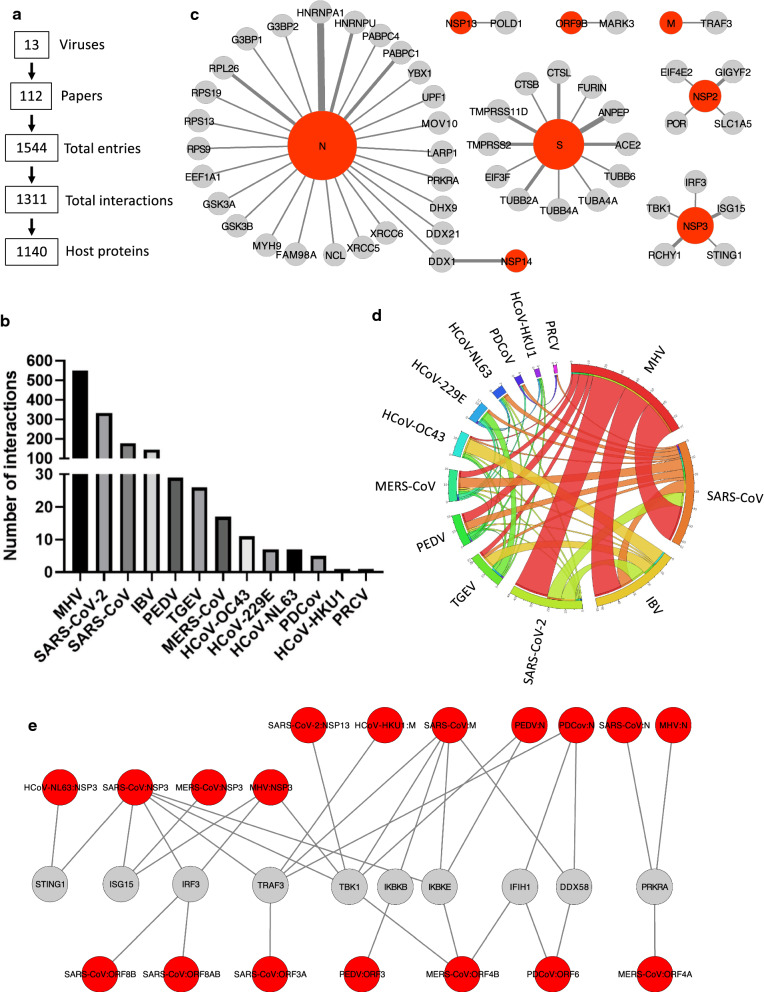
Quantitative analysis of collected virus host-interactions. **a** Key numbers describing the database that has been assembled. **b** Numbers of distinct interactions that have been collected for each virus. **c** Orthologous interactions conserved between several viruses. The thickness of the lines is proportional to the number of viruses for which the interaction was reported. Displayed graph was generated using Cytoscape [79]. **d** Circular diagram showing the proportion of shared host protein targets between analyzed coronaviruses. Display was obtained using the Circos table viewer [80]. **e** Innate immunity factors interacting with several coronavirus proteins

We collected virus-host interaction data from literature for the 13 coronaviruses mentioned above (*i.e.* HCoV-NL63, HCoV-229E, HCoV-HKU1, HCoV-OC43, MERS-CoV, SARS-CoV, SARS-CoV-2, TGEV, PRCV, PEDV, MHV, IBV, and PDCoV), and 112 publications explicitly reporting physical interactions between viral and host proteins were identified (Fig. 1a). Their analysis allowed us to collect 1544 entries for virus-host PPIs that were gathered in a single data file (Additional file 1: Table S1). As 133 interactions were characterized by more than one method or were detected across multiple host species, and that 23 interactions were reported in two or more independent publications, this corresponds to 1311 distinct virus-host interactions involving 1140 different host proteins (orthologous proteins from different host species were collapsed; Fig. 1a and Additional file 1: Table S2). A majority of the reported interactions (92%) were from four viruses for which high-throughput interactomic methods have been applied: MHV, SARS-CoV-2, SARS-CoV, and IBV (Fig. 1b, Additional file 1: Table S3) [3, 14–20].

### Shared interactions and host protein targets across multiple coronaviruses

We then looked for interactions that are conserved across two or more viruses. To perform this analysis, orthologous proteins from different host species were collapsed as above. In total, we identified 51 orthologous interactions in the dataset, providing a robust network of shared PPIs between several coronaviruses (Fig. 1c; Additional file 1: Table S4). Membrane receptors shared by several coronaviruses (ACE2, ANPEP) as well as cellular proteases involved in the processing of the spike glycoproteins S (TMPRSS2, TMPRSS11D, CTSB, CTSL, FURIN) were highlighted. Multiple interactions between the nucleoprotein N and host factors involved in mRNA synthesis, maturation, nuclear export, translation and stability were also identified (EEF1A1, PABPC1, PABPC4, RNRNPA1, HNRNPU, YBX1, LARP1), including ribosomal components (RPS9, RPS13, RPS19, RPL26), multiple helicases (UPF1, MOV10, DHX9, DDX21, DDX1) and key components of the innate antiviral response (G3BP1, G3BP2, PRKRA). GSK3A/B were previously reported to phosphorylate the viral nucleocapsid of MHV to recruit DDX1 [21]. In the assembled interactome dataset, GSK3A/B were also found to interact with N of IBV and SARS-CoV, and interaction of DDX1 with both N and nsP14 of IBV and TGEV as well as nsP14 of SARS-CoV were also reported [20, 22–24]. This analysis also highlights the well-known function of nsP3 in coronavirus escape from the innate immune response through interactions with both ubiquitination/ISGylation factors (RCHY1, ISG15) and key antiviral factors (STING1, TBK1, IRF3) [19, 25–29]. Other remarkable interactions are between nsP2, EIF4E2 (4EHP) and GIGYF2, which are two components of a complex repressing mRNA translation [30]. Although EIF4E2 and GIGYF2 were identified by high-throughput interactomic applied to SARS-CoV, SARS-CoV-2 and MHV [3, 14, 17], they were never investigated in details. These highly conserved interactions suggest a crucial role of nsP2 in regulating viral and/or cellular mRNA translation and degradation.

We also looked for host proteins interacting with several coronaviruses, but not necessarily targeted by the same viral proteins. Because coronavirus proteins assemble into large molecular complexes, the same host protein can be captured, either directly or indirectly, by distinct viral proteins used as bait. In total, 105 of the 1140 host proteins from the dataset (9.2%) were shared by at least two coronaviruses, including 50 host proteins involved in the conserved interactions displayed in Fig. 1c. Shared targets of different viruses are illustrated in Fig. 1d and details are provided in Additional file 1: Table S5. Interestingly, several host factors involved in viral RNA sensing (DDX58, IFIH1, PRKRA), DNA sensing (STING1) or downstream signalling to activate the innate immune response (TRAF3, TBK1, IKBKE, IKBKB, ISG15, IRF3) were targeted by unrelated viral proteins (Fig. 1e) [3, 28, 29, 31–44]. This highlights the importance of these interactions for coronaviruses, but is puzzling from an evolutionary perspective. Indeed, this suggests that coronaviruses have evolved different but convergent strategies to target these innate immunity factors. An alternative explanation is that multiple proteins of a virus target the same host factor in redundant ways, although this information is not present in the current interaction datasets yet. The two hypotheses are not mutually exclusive and this will require further investigation to be addressed.

### Enrichment analysis for specific cellular pathways

**Fig. 2:**
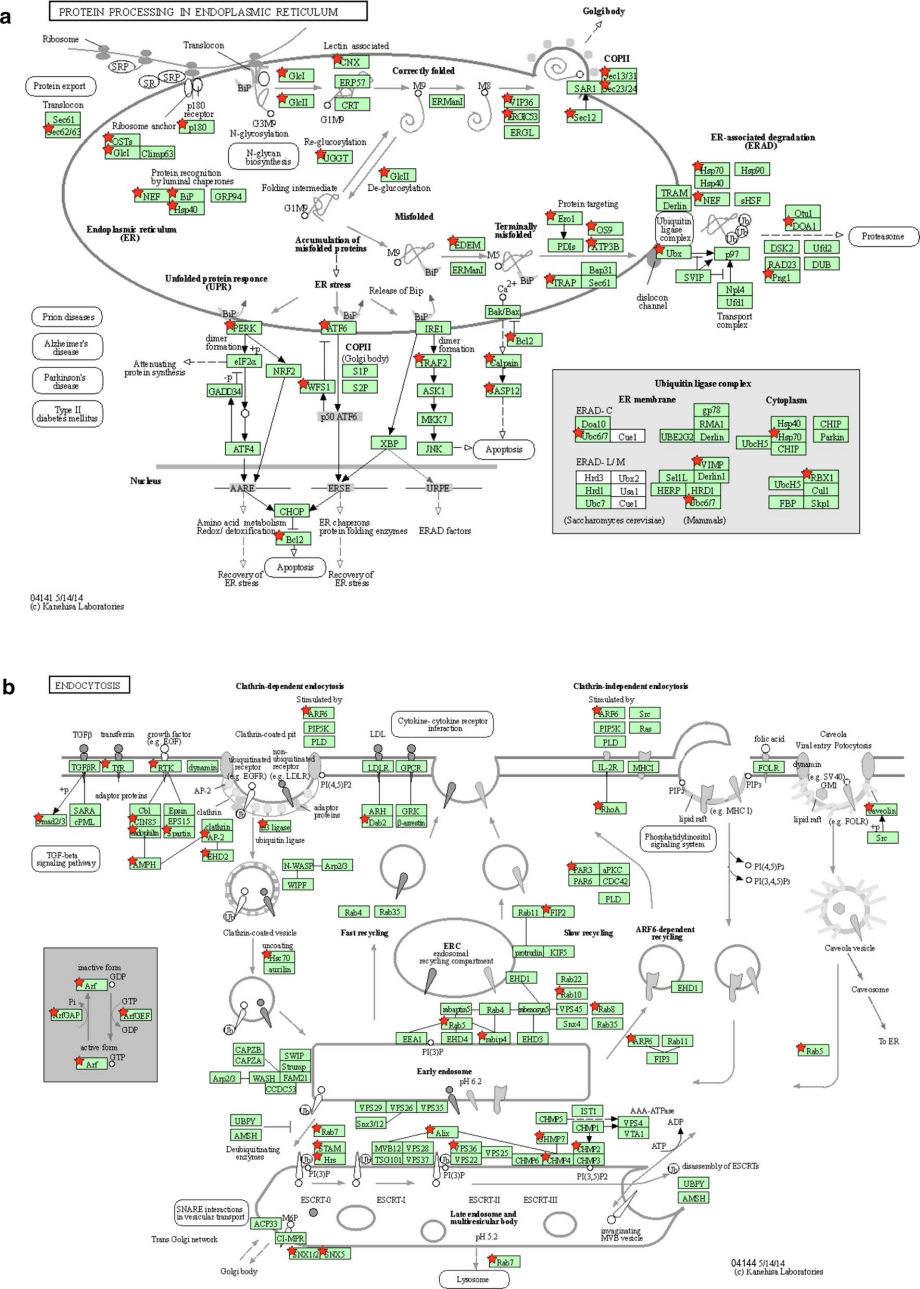

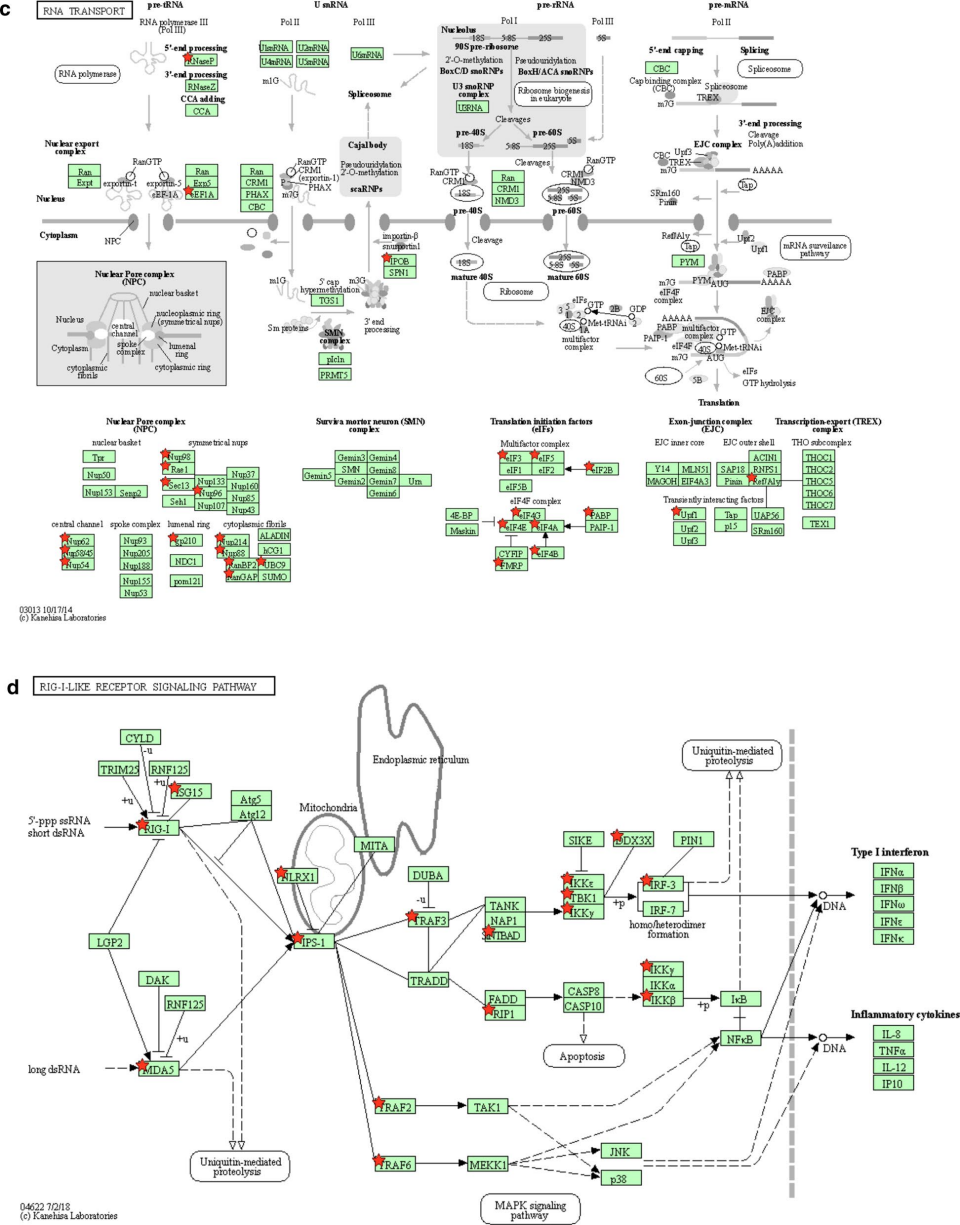
KEGG pathways enriched in the list of coronavirus-interacting proteins. **a** Protein processing in endoplasmic reticulum (KEGG map ID: 04141). **b** Endocytosis (KEGG map ID: 04144). **c** RNA transport (KEGG map ID: 03013). **d** RIG-I-like receptor signaling (KEGG map ID: 04622). Host proteins interacting with coronavirus proteins are marked with red stars

To identify cellular complexes or pathways that are enriched in the list of host factors interacting with coronavirus proteins, we retrieved associated KEGG annotations and calculated statistical enrichments using the DAVID functional annotation tool [6, 7]. To evaluate the interest of combining interactome data from several coronaviruses, we analyzed host targets of SARS-CoV-2 alone (Table 1a), of human highly-pathogenic coronaviruses (Table 1b) and of all coronaviruses (Table 1c). Although SARS-CoV-2 restricted analysis only identified “Protein processing in endoplasmic reticulum” and “RNA transport” as significantly enriched KEGG pathways, expending the dataset doubled the number of host factors falling in these two categories and unraveled enrichments for “Ribosome”, “RIG-I-like receptor signaling”, “Endocytosis”, “Spliceosome” and “Phagosome” pathways (Table 1c and Additional file 1: Table S6). Up to 68% of the KEGG-annotated eukaryotic ribosomal proteins and 18% of the spliceosome components are targeted by coronavirus proteins, thus demonstrating the importance for these viruses to control the mRNA processing and translation machinery. We used KEGG Mapper to highlight targeted host proteins on schematic representation of the most enriched cellular pathways (Fig. 2a-d) [45]. As shown in Fig. 2a, several factors involved in protein export were targeted together with components of the unfolded protein response/endoplasmic-reticulum-associated protein degradation (ERAD) pathway. This observation is in line with numerous reports showing that coronavirus replication induces a strong ER stress response, and that virulence factors expressed by these viruses aim at controlling and even subvert this cellular response to assemble convoluted and double-membrane vesicles [46]. Several regulators of intracellular trafficking and vesicular transport associated to endocytic compartments, especially late endosomes and multivesicular bodies, are also hijacked (Fig. 2b). These interactions most likely contribute to the entry, assembly and/or secretion of viral particles [47]. A statistical enrichment of the KEGG annotation “RNA transport” was also observed because components of the nuclear pore complex (NPC) and translation initiation factors, which take in charge mRNA after their transport through the nuclear pore, are also overrepresented in the list of host proteins targeted by coronaviruses (Fig. 2c). The number of Interactions with translation initiation factors, together with ribosomal components, reflects the intense hijacking of translational machinery by coronaviruses [14]. Some of these interactions could also prevent the translational shut-off associated to antiviral and unfolded protein responses as demonstrated for the ORF7 of TGEV [48], while others could help the virus to control host protein expression [49]. Consequences of viral factors interacting with components of the NPC are poorly documented [50], but interference mechanisms with the innate antiviral response have been suggested [51]. Whether these interactions prevent the nuclear import of transcription factors or the nuclear export of cellular mRNA to blunt the immune response as shown for other viruses should be explored [52]. Finally, this analysis highlighted multiple interactions with components of the RIG-like receptor pathways (Fig. 2d), that is essential for viral RNA detection and the induction of interferon secretion. This includes the multi-targeted host factors already presented in Fig. 1e but also additional proteins such as the adaptors MAVS (IPS1), TBKBP1 (SINTBAD), TRAF2 and TRAF6, the negative (NLRX1) or positive (DDX3X) regulators, and kinases such as RIPK1 (RIP1) or IKBKG (IKKγ) [3, 20, 53, 54]. Some of these interactions probably reflect the sensing of coronavirus components by immune receptors which trigger the antiviral response, whereas others correspond to countermeasures that coronaviruses evolved to escape this response [55]. Altogether, this provides an overview of cellular pathways that are major targets of coronaviruses.

**Table 1:** KEGG pathways enrichment in the list of host factors interacting with viral proteins for SARS-CoV-2 (**a**), highly pathogenic hCoVs (**b**) and all coronaviruses (**c**)

Kegg pathway (Term)	Count	%	p-value	Fold enrichment	Benjamini p-value
**a—Kegg pathways enrichment in SARS-CoV-2 host protein targets (n = 333)**					
Protein processing in endoplasmic reticulum	13	4	8.80E−05	4	1.70E−02
RNA transport	12	3.7	4.40E−04	3.6	4.10E−02
**b—Kegg pathways enrichment in highly pathogenic hCoVs host protein targets (n = 483)**					
RIG-I-like receptor signaling pathway	14	3	1.30E−07	6.6	3.00E−05
NF-kappa B signaling pathway	13	2.8	1.10E−05	4.9	8.00E−04
RNA transport	19	4.1	4.10E−06	3.6	4.50E−04
Protein processing in endoplasmic reticulum	18	3.9	1.30E−05	3.5	7.20E−04
Epstein-Barr virus infection	13	2.8	3.10E−04	3.5	1.10E−02
Influenza A	18	3.9	1.90E−05	3.4	8.40E−04
Measles	13	2.8	6.80E−04	3.2	2.10E−02
**c—Kegg pathways enrichment in Coronaviruses host protein targets (n = 1140)**					
Ribosome	59	5.4	9.0E−31	5.8	2.3E−28
RIG-I-like receptor signaling pathway	16	1.5	1.60E−04	3.1	5.60E−03
Protein processing in endoplasmic reticulum	37	3.4	5.20E−09	2.9	6.60E−07
RNA transport	33	3	1.00E−06	2.6	6.30E−05
Endocytosis	43	3.9	1.30E−07	2.4	1.10E−05
Spliceosome	24	2.2	1.10E−04	2.4	4.70E−03
Phagosome	26	2.4	1.00E−04	2.3	5.30E−03

**Fig. 3:**
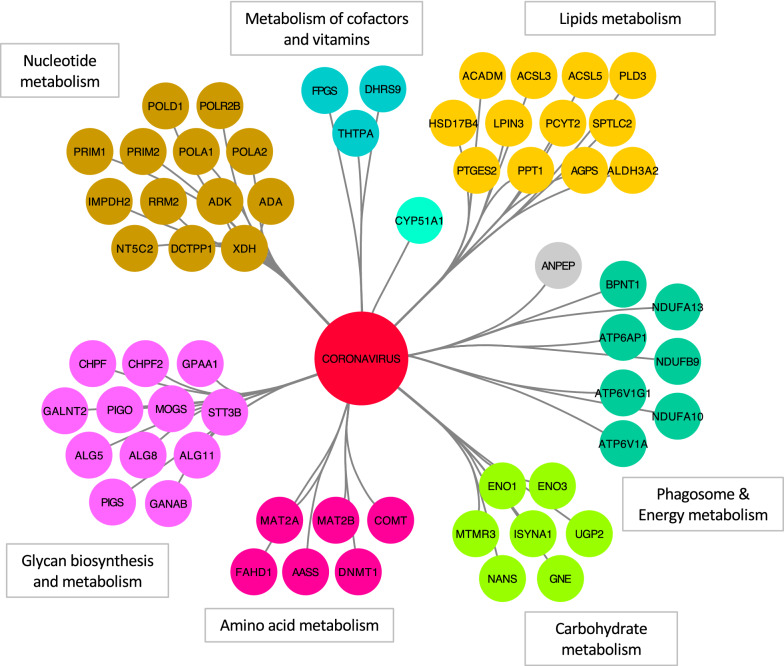
Interactions of coronavirus proteins with metabolic pathways. Coronavirus-interacting proteins that are involved in cellular metabolism are displayed (i.e. with the KEGG tag “metabolic pathway”). Host factors were clustered and colored according to the specific pathways they belong to using KEGG annotation

Despite several reports showing that viruses have evolved strategies to finely tune cellular metabolism for promoting their replication [56], no statistical enrichment for components of a specific metabolic pathway was identified. We thus conducted an orientated analysis by looking at potential overlaps between the list of host proteins targeted by coronaviruses and the proteins annotated in the “Metabolic pathways” of the KEGG database. In total, 62 proteins were identified at the intersection of the two lists, and were clustered according to their specific metabolic pathways using KEGG annotation (Fig. 3). Multiple interactions were identified with factors involved in nucleotide metabolism, including enzymes of the nucleo-tide/-side biosynthesis and degradation pathways (IMPDH2, RRM2, ADK, DCTPP1, NT5C2, XDH, ADA) and more surprisingly, several components of cellular DNA and RNA polymerases (POLA1, POLA2, POLD1, PRIM1, PRIM2 and POLR2B). These interactions could contribute to the DNA replication stress and cell cycle arrest induced by coronaviruses as previously suggested for the nsP13-POLD1 interaction, but their role is still poorly defined [57]. Several components of complex I of the mitochondrial respiratory chain were also targeted (NDUF9/10/13), as well as V-type ATPase subunits involved in the acidification of cellular compartments, especially during phagocytosis (Additional file 1: Table S6). Lipid and glycan biosynthesis enzymes were highly represented, in line with previous reports showing the dependence of coronaviruses to these two pathways [58, 59]. In the “amino acid metabolism” cluster, both methionine adenosyltransferase 2A (MAT2A) and the regulatory subunit MAT2B were present (captured by ORF3 from PEDV and nsP9 of SARS-CoV-2, respectively). MAT2A catalyzes the synthesis of S-adenosyl-l-methionine, the major biological methyl donor. This may indicate that coronaviruses critically need S-adenosyl methionine (SAM) to methylate the viral RNA cap structures to allow transcription and prevent their recognition by cellular innate immunity receptors [60]. In the same cluster were also found two methyltransferases (COMT, DNMT1). Although the significance of targeting COMT (Catechol O-Methyl Transferase), an enzyme involved in catecholamine synthesis, remains elusive, interactions with DNMT1 could be a mechanism used by coronaviruses to alter the epigenetic landscape of infected cells as observed for other viruses [61]. Finally, coronavirus proteins also interacted with several host factors flagged as “central carbon metabolism” enzymes. This cluster includes multiple enzymes contributing to protein sialylation (GNE, NANS) and glycosylation (UGP2), inositol synthesis (MTMR3, ISYNA1), and two enolases (ENO1, ENO3) whose interactions with viral proteins could modulate glycolysis and energy supply for promoting viral growth. Altogether, this analysis suggests that, although the completeness of the available dataset does not allow statistical validation, coronaviruses extensively interact with metabolic enzymes.

### A network-based identification of host protein complexes targeted by coronaviruses

**Fig. 4:**
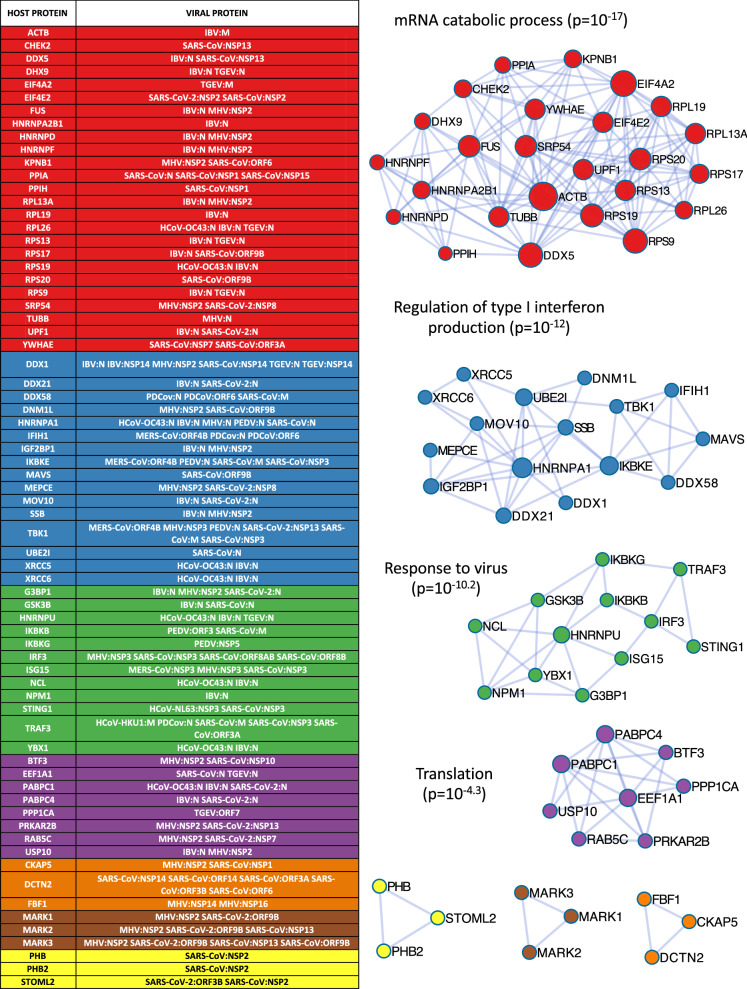
Interactions of coronavirus proteins with clusters of tightly connected proteins in the human interactome. We established a core list of host proteins for which multiple experimental evidences exist to support an interaction with coronavirus proteins. This includes virus-host interactions validated by different technics in one report or confirmed across multiple publications. Host proteins captured independently by different viral proteins were also included. Metascape was used to identify the seven clusters that are presented (blue lines indicate PPIs from the human interactome). Supporting virus-host interactions are detailed in the left table. Functional annotation tool integrated to Metascape was used to determine most statistically enriched GO terms (“Biological Process”) and annotate the clusters (p-values are indicated)

To identify densely connected regions in the list of targeted host proteins, we used the web-based application Metascape [8]. As no result could be retrieved when the full list of 1140 host proteins was submitted to Metascape, we limited this analysis to the core list of 178 host proteins for which multiple experimental evidences exist to support an interaction with coronavirus proteins. We also excluded the interactors of the spike glycoproteins that are already well-characterized and skewed the analysis of the other virus-host PPIs (Additional file 1: Table S7). From the list of 156 host proteins, this identified 7 protein clusters whose biological role was determined by Metascape using GO enrichment analysis (Fig. 4) [62, 63] and largely confirmed our analyses discussed above. The functional annotations enriched in the four largest clusters were “mRNA catabolic process”, “Regulation of type I interferon production”, “Response to virus”, and “Translation”. These interaction modules partially overlap and well-complement two of the KEGG pathways pinpointed by the enrichment analysis described above, i.e. “RNA transport” and “RIG-I-Like Receptor Signaling Pathway”, respectively (Fig. 2c, d; Additional file 1: Table S6). The “mRNA catabolic process” cluster contains four proteins involved in KEGG’s “RNA transport pathway”. This includes UPF1 that is involved in nonsense-mediated decay of mRNAs containing premature stop codons, EIF4A2 and EIF4E2 that repress mRNA translation, and the importin subunit KPNB1. Interestingly, this analysis also highlighted the link between XRCC5/6 dimers and components of the RIG-like receptor pathway. XRCC5/6 binds to DNA double-strand break ends and activates the catalytic activity of DNA-PK to promote DNA repair. In the cytosolic compartment, it also participates to type I interferon induction in response to foreign DNA [64]. Furthermore, it has been shown that DNA-PK is activated and participates to interferon induction in dengue virus-infected cells [65], and that NS5A from Hepatitis C virus (HCV) is phosphorylated by this kinase [66]. The targeting of XRCC5 and 6 by the N protein of IBV and OC43 [20, 67] suggests that these proteins also play a role in the induction of innate immunity and/or the replication of coronaviruses. Three clusters of only three components were also identified. The first one includes PHB, PHB2 and STOML2 that are all members of the stomatin-prohibitin-flotillin-HflC/K (SPFH) superfamily. These proteins colocalize at the inner mitochondrial membrane where they assemble into ring-like structures [68]. Cornillez-Ty CT. et al. previously highlighted interactions of SARS-CoV nsP2 with prohibitins PHB and PHB2 [17], and STOML2 was also present in their dataset. Interestingly, the recent report of Gordon et al. also identified an interaction of ORF3B from SARS-CoV-2 with STOML2 [3]. These cellular proteins regulate mitochondrial homeostasis, and are involved in processes such as mitophagy and mitochondrial fusion. By targeting these proteins, coronavirus proteins may also impact key mitochondrial functions such as respiration but also lipid homeostasis and innate immunity. Indeed, mitochondria are involved in both lipogenesis and lipolysis, and prohibitin expression has been shown to impact lipid accumulation and degradation [69, 70]. It should be determined if such interactions with prohibitins contribute to the massive remodeling of intracellular lipid membranes induced by coronavirus infections. As prohibitins also regulate mitochondrial fusion and fission, for which the impact on antiviral-signaling has been well documented [71], this could have indirect consequences on the antiviral response. In addition, recent studies have established physical interactions between components of the prohibitin complex and MAVS, a pivotal adaptor in viral RNA sensing and interferon induction [72]. The second small cluster is composed of MARK1, 2, and 3, three Ser/Thr protein kinases involved in the control of cell polarity, microtubule stability and cancer. MARK proteins have been reported to interact with nsP2 of MHV, nsP13 of SARS-CoV, and ORF9B of both SARS-CoV and SARS-CoV-2 [3, 14–16]. Although their role in coronavirus replication is unknown, they could contribute to viral trafficking as they control microtubule dynamics and vesicular transport. Interestingly, several approved drugs inhibiting MARK kinases were recently proposed as potential antivirals against coronaviruses and especially SARS-CoV-2 [3]. The last cluster is composed of the cytoskeleton and microtubule-associated proteins DCTN2, FBF1 and CKAP5 that could participate to the transport of viral complexes within infected cells.

### Overlaps with functional in vitro screenings of chemical drug libraries

**Fig. 5:**
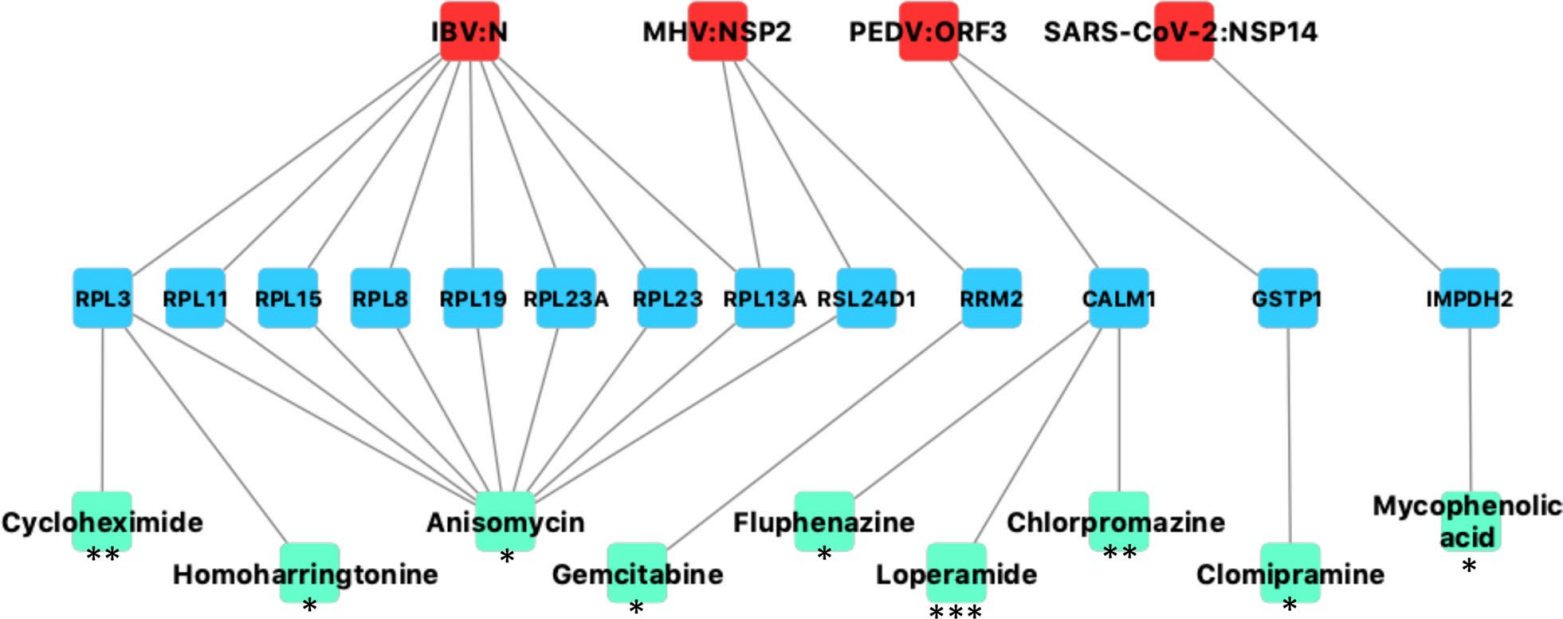
Intersection between the coronavirus-host interactome and cellular targets of drugs inhibiting coronavirus replication. Viral proteins are in red, host proteins in blue and small molecules in cyan. Virus-host PPIs are from the coronavirus-host interactome. Compound-target interactions were retrieved from the Drug Repurposing Hub database. For each molecule, stars indicate how many screenings out of the four compiled for this analysis identified their anti-coronavirus activity

To provide a rational for the antiviral activity of some drugs targeting cellular factors, we determined to what extent the coronavirus-host interactome intersects the large-scale chemical screenings of antivirals against coronaviruses. We selected four publications reporting in vitro screening of chemical libraries against SARS-CoV, MERS-CoV and HCoV-OC43 [10–13]. In total, 77 molecules were identified for their antiviral activity against at least one coronavirus. Chloroquine was identified in all four publications, amodiaquine and loperamide were identified in three of them, and chlorpromazine, cycloheximide, emetine, and hydroxychloroquine were cited in two publications. Then we searched for cellular targets of these 77 molecules using the Drug Repurposing Hub database [9], and looked for overlaps with the list of 1140 host proteins interacting with coronaviruses. We identified 9 drugs and 13 host proteins matching these criteria (Fig. 5). Anisomycine, cycloheximide and homoarringtonine are translational inhibitors interacting with ribosomal components. As coronaviruses exhibit tight interactions with translation initiation factors and recruit multiple ribosomal components (Table 1c and Fig. 4), this could explain the antiviral effect of these three drugs. Three molecules, fluphenazine, loperamide and chlorpromazine, target calmodulin (CALM1) which interacts with ORF3 from PEDV [42]. CALM1 is a Ca^2+^-binding messenger protein that regulates the function of numerous proteins. In particular, it controls the activity of the multifunctional CAMKII proteins (Ca^2+^/calmodulin-dependent protein kinase II), which are targeted by coronaviruses as well. Indeed, CAMK2D and CAMK2G interact with nsP3 of SARS-CoV and nsP2 of MHV, respectively (Additional file 1: Table S1) [14, 19]. Furthermore, ACE2 receptor of SARS-CoV-2, SARS-CoV, HCoV-NL63 and HCoV-229E, and CEACAM1 receptor of MHV are also calmodulin-binding proteins [73–75]. Although fluphenazine, loperamide and chlorpromazine have multiple targets in host cells, interactions with CALM1 could account for the inhibition of coronaviruses by these drugs. Clomipramine, which is structurally and functionally closely related to chlorpromazine, was also selected in the analysis as a consequence of its interaction with GSTP1, one of the glutathione S-transferase isoenzymes. This analysis also highlighted interactions between coronavirus proteins and host factors involved in nucleotide biosynthesis. First, nsP14 interacts with inosine-5′-monophosphate dehydrogenase 2 (IMPDH2), a key enzyme of de novo purine biosynthesis pathway. The inhibitor mycophenolic acid, that is depleting cells in purine, is well-known for its broad-spectrum antiviral activity and is in a list of drug candidates proposed against SARS-CoV-2 [3]. In addition, the analysis pinpointed to the interaction of nsP2 from MHV with the host enzyme RRM2, one of the two subunits of ribonucleotide reductase that is inhibited by gemcitabine. This interaction between an enzyme involved in deoxyribonucleotide synthesis and an RNA virus protein was unexpected. However, RRM2 was previously reported to interact with NS5B from HCV, preventing its degradation and promoting the replication of this RNA virus [76]. Furthermore, gemcitabine has been shown to inhibit the replication of many different RNA viruses by blocking cellular DNA replication. Indeed, this induces a genomic stress that triggers the innate antiviral response [77]. Thus, RNA viruses could have evolved interactions with cellular enzymes involved in nucleoside/nucleotide synthesis such as IMPDH and RRM2 to sustain DNA replication and prevent genotoxic stress.

## Concluding remarks

We have assembled a large coronavirus-host interactome built upon 1311 PPIs retrieved from literature. Functional annotation and network-based analyses highlighted targeted cellular pathways and modules. Overall, mRNA processing and transport, translation initiation and protein translation, endosomal trafficking, and innate immunity were the most enriched pathways. This conclusion is consistent with previous reports identifying these biological processes as targets of specific coronaviruses. The global analysis suggest that these interactions are highly conserved across most if not all coronaviruses. We also pinpointed to a couple of small protein complexes that appear particularly relevant to coronavirus infection but were not previously investigated. This includes in particular the EIF4E2-GIGYF2 dimer involved in the repression of protein translation, the MAT2A-MAT2B complex controlling SAM synthesis, the DNA-PK kinase that contributes to interferon induction, and the mitochondrial proteins PHB, PHB2 and STOML2 regulating mitophagy. Multiple evidences also support a key role of the MARK kinases. Finally, we identified a dozen of host factors that are bound by coronavirus proteins and functionally modulated by compounds selected from antiviral screens, giving hints to explain the inhibition of coronavirus replication by these drugs. Among these molecules is chlorpromazine, an antipsychotic drug that is currently evaluated in a clinical trial against SARS-CoV-2 [78]. In conclusion, this work has highlighted the importance of several neglected coronavirus-host interactions that deserve to be further investigated. It also illustrates the interest of combining virus-host interactome datasets from different laboratories, obtained by different approaches and generated in various cell types to increase coverage and get closer to completeness.

## Supplementary information

**Additional file 1. Table S1:** Complete list of coronavirus-host interaction data retrieved from literature.** Table S2: **Consolidated, non-redundant list of coronavirus-host protein-protein interactions.** Table S3:** Number of interactions identified for each coronavirus.** Table S4:** List of interactions reported multiple times accross different coronaviruses (i.e. orthologous interactions).** Table S5:** Matrix of host proteins interacting with multiple coronaviruses.** Table S6:** Host proteins present in enriched KEGG pathways from Table 1c.** Table S7:** Consolidated, non-redundant list of host proteins present in Table S1 with or without interactors of S (left and right columns, respectively).

## Data Availability

All data generated or analyzed during this study are included in this published article and its additional file.

## Abbreviations

SARS-CoV: Severe acute respiratory syndrome coronavirus
MERS-CoV: Middle East respiratory syndrome-related coronavirus
PPIs: Protein–Protein Interactions
HCoV: Human coronavirus
TGEV: Transmissible gastroenteritis virus
PRCV: Porcine respiratory coronavirus
PEDV: Porcine epidemic diarrhea virus
MHV: Murine hepatitis virus
IBV: Infectious bronchitis virus
PDCoV: Porcine deltacoronavirus
nsPs: Non-structural proteins
ERAD: Endoplasmic-reticulum-associated protein degradation
ER: Endoplasmic reticulum
